# Investigating the pathophysiology and evolution of internal carotid dissection: a fluid–structure interaction simulation study

**DOI:** 10.3389/fneur.2024.1455989

**Published:** 2024-09-30

**Authors:** Adriano Bonura, Giulio Musotto, Gianmarco Iaccarino, Sergio Soeren Rossi, Rosalinda Calandrelli, Fioravante Capone, Vincenzo Di Lazzaro, Fabio Pilato

**Affiliations:** ^1^Research Unit of Neurology, Department of Medicine and Surgery, Università Campus Bio-Medico di Roma, Rome, Italy; ^2^Fondazione Policlinico Universitario Campus Bio-Medico, Rome, Italy; ^3^Bioengineering Unit, Ri.MED Foundation, Palermo, Italy; ^4^Radiology and Neuroradiology Unit, Department of Imaging, Radiation Therapy and Hematology, Università Cattolica del Sacro Cuore, Fondazione Policlinico Universitario Agostino Gemelli, Istituto di Ricovero e Cura a Carattere Scientifico (IRCCS), Rome, Italy

**Keywords:** stroke, fluid dynamics, internal carotid dissection, fluid–structure interaction, computer simulation

## Abstract

**Background:**

Arterial dissection, a condition marked by the tearing of the carotid artery’s inner layers, can result in varied clinical outcomes, including progression, stability, or spontaneous regression. Understanding these outcomes’ underlying mechanisms is crucial for enhancing patient care, particularly with the increasing use of computer simulations in medical diagnostics and treatment planning. The aim of this study is to utilize computational analysis of blood flow and vascular wall to: (1) understand the pathophysiology of stroke-like episodes in patients with carotid artery dissection; and (2) assess the effectiveness of this method in predicting the evolution of carotid dissection.

**Methods:**

Utilizing contrast-enhanced magnetic resonance angiography (MRA), we segmented images of the patient’s right internal carotid artery. These images were transformed into 3D solids for simulation in Ansys multifisic software, employing a two-way fluid structure interaction (FSI) analysis. Simulations were conducted across two wall conditions (atherosclerotic and normal) and three pressure states (hypotension, normotension, hypertension).

**Results:**

The simulations indicated a significant pressure discrepancy between the true and false lumens of the artery. This suggests that flap motion and functional occlusion under hypertensive conditions could be the cause of the clinical episodes. Thrombotic risk and potential for dissection extension were not found to be critical concerns. However, a non-negligible risk of vessel dilation was assessed, aligning with the patient’s clinical follow-up data.

**Conclusion:**

This study highlights specific hemodynamic parameters that could elucidate carotid artery dissection’s mechanisms, offering a potential predictive tool for assessing dissection progression and informing personalized patient care strategies.

## Introduction

Arterial dissection refers to a tear or rupture in the inner layers of the carotid artery, resulting in a change in vessel morphology and blood flow (1). While arterial dissection can affect any artery, leading to blood clot formation, artery narrowing, and even complete blockage of blood flow, the involvement of extracranial neck arteries and intracranial arteries is particularly significant because it can cause ischemic stroke (1). Dissection beginning in the vessel’s inner wall, may also extend to the outer wall and both mechanisms can lead to the obstruction of downstream circulation, causing tissue hypoperfusion and, consequently, major ischemic events and strokes when the internal carotid artery (ICA) is involved (1–3). Additionally, the vessel wall becomes more fragile, increasing the risk of dilatation, rupture, and bleeding (4, 5). While traumatic carotid dissections result from direct injury or trauma to the neck (1), arterial dissections may occur without any apparent cause, namely spontaneous dissection, and may be associated with connective tissue disorders, genetic factors, or spontaneous activities like sneezing, coughing, or physical activity (1).

Non-invasive neuroimaging has a pivotal role in the diagnosis of carotid artery dissections by visualizing vessels’ wall and blood flow by color-doppler ultrasound (DUS), computed tomography angiography (CTA), magnetic resonance angiography (MRA), and in selected cases, digital subtraction angiography (DSA) (5).

The natural history and outcome of carotid dissection are extremely variable. The dissection may progress and expand, remain stable, or regress spontaneously with the rejoint of the vessel wall layers, but its determinants are not fully understood (6, 7).

In the medical field, computer-based technologies have been integrated into various medical fields, offering guidance to clinicians in their clinical decision-making processes (8). In particular, computer simulation entails the utilization of computer-based models to replicate genuine medical scenarios, serving the purposes of training, education, research, and patient care (9).

Recently, artificial intelligence (AI) and software advancements have enabled the use of PC-based or simulation systems to virtualize pathological conditions (10, 11).

In particular, computational analysis can provide extremely valuable insights in the cardio-cerebrovascular field through methodologies such as Computational Fluid Dynamics (CFD) analysis and Fluid–Structure Interaction (FSI) analysis. The latter, in particular, allows the study of the interaction between blood flow and the vascular or cardiac wall, enabling a thorough and highly personalized patient assessment. This approach also offers predictive and prognostic information.

In particular, computational analysis can provide highly valuable insights in the cardio-cerebrovascular field through methodologies such as Computational Fluid Dynamics (CFD) analysis and Fluid–Structure Interaction (FSI) analysis. In the medical field, CFD allows the study of blood flow and detailed analysis of associated rheological parameters (12). FSI, on the other hand, enables the investigation of the interaction between blood flow and the vascular or cardiac wall (13), ensuring a thorough and highly personalized patient assessment (14–20). This approach also offers predictive and prognostic information (19, 21).

We employed FSI analysis to examine the mechanical and rheological characteristics of symptomatic arterial dissection in a patient with a symptomatic right internal carotid artery (ICA) dissection, who exhibited neurological symptoms during episodes of elevated blood pressure. The objective of our study was to comprehend the pathophysiological mechanisms underlying neurological symptoms and analyze parameters that could aid in predicting the evolution of the dissection.

### Case description

A 75-year-old patient with a medical history of hypertension, hypercholesterolemia, and bronchial asthma, receiving treatment with angiotensin receptor blockers, was brought to the emergency department due to the sudden onset of left-sided weakness and dysarthria during a hypertensive crisis (blood pressure 180/100 mmHg). Interestingly, when the patient stood up in the emergency room, blood pressure dropped, and neurological symptoms improved. After a few hours, the blood pressure rose again (190/100 mmHg), and neurological symptoms returned. This cycle repeated several times during emergency room stay.

**Figure 1:**
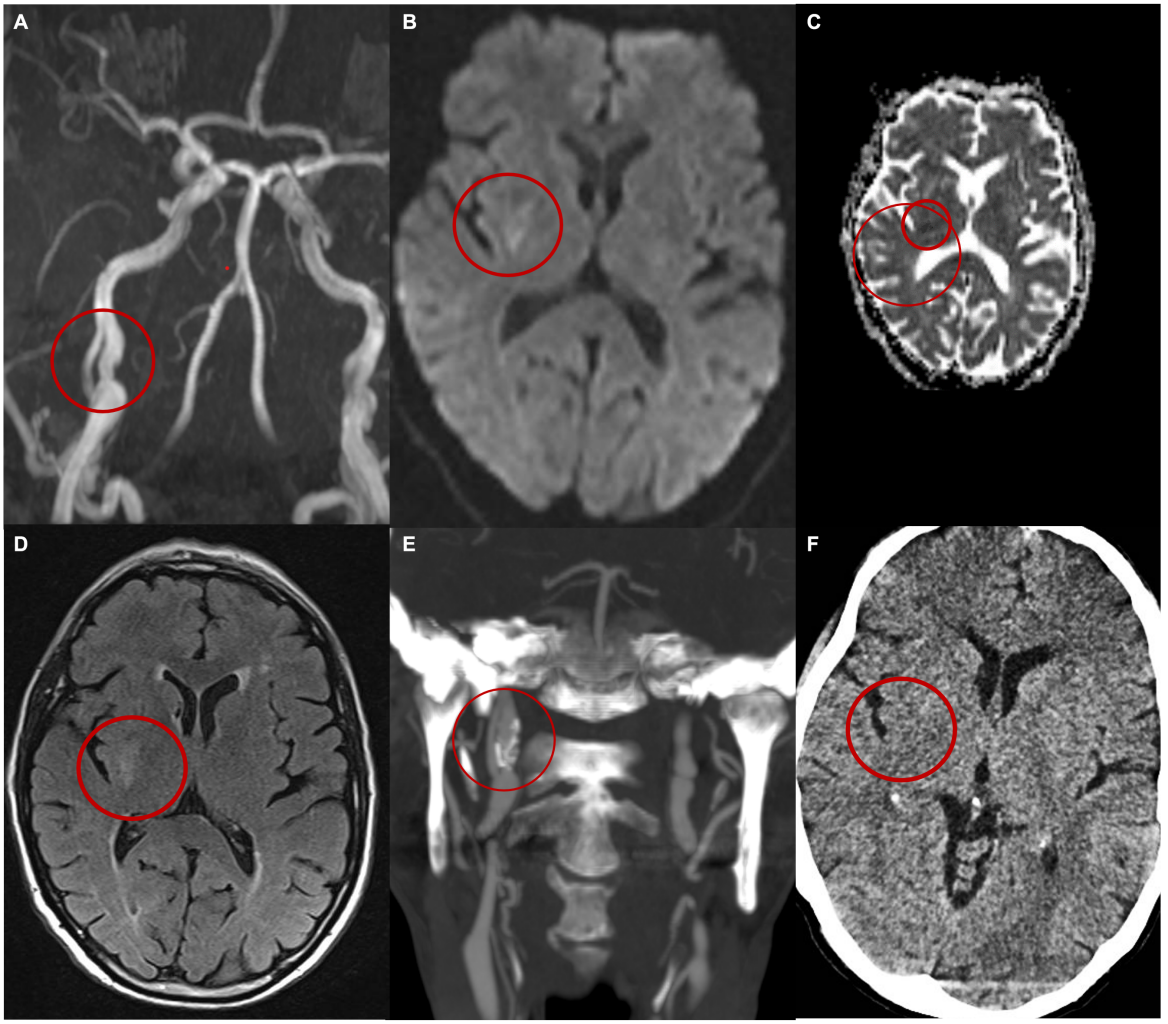
Patient’s imaging. **(A)** MIP MR angiography with gadolinium images reveals a dissection of the right internal carotid artery characterized by the presence of a double lumen and an intimal flap. **(B)** MRi DWI shows right capsular hyperintensity compatible with an acute ischaemic lesion of the right capsule. **(C)** ADC shows restriction of the diffusivity. **(D)** MRi FLAIR. Images at the 6-month follow-up **(E)** shows on the CT angiography the persistence of a double lumen and the presence of an aneurysmal dilation in the true lumen **(E)**. **(F)** CT brain at 6 months.

A brain CT scan with CTA was performed, revealing a dissection of the right ICA in the extracranial segment 3 cm from its origin, without evidence of acute ischemic or haemorrhagic lesions. Notably, the neurological symptoms regressed during examination. The patient was started on treatment with acetylsalicylic acid, atorvastatin, and antihypertensive therapy and was subsequently admitted to the stroke unit. DUS confirmed a double lumen affecting the right ICA. Further evaluation with brain MRI including 3D-angio sequences with gadolinium confirmed the double lumen at the C2 to C3 segment of the right ICA revealing evidence of an intramural hematoma upstream of the dissection (Figure 1A). Additionally, a subacute lesion was found in the right putamen on diffusion-weighted imaging, apparent diffusion coefficient and fluid-attenuated inversion recovery sequences (Figures 1B–D).

The patient was discharged home after a 5-day hospital stay without any neurological symptoms. A CT angiography conducted 6 months post-discharge revealed the persistence of the dissection with an intimal flap, the presence of an aneurysmal dilation of the medial wall of the vessel, and calcifications of the vessel walls (Figure 1E). Throughout the 6-month follow-up period, the patient did not experience any additional neurological symptoms and any new lesions at CT imaging (Figure 1F).

### Introduction to fluid dynamics principle in vascular system

Fluid dynamics applied to medicine primarily focuses on studying key parameters such as shear strain rate, shear stress, and blood viscosity.

The shear strain rate (or shear rate) represents the gradient among the velocities of the different fluid layers (22). In a laminar regime, the flow in the longitudinal section exhibits a parabolic shape, with the central layers moving faster than the outer layers near the wall. As the shear rate increases, the velocity difference between these layers also increases, leading to a more pronounced parabolic flow (22). Conversely, shear stress represents the tangential force of the flowing blood on the endothelial surface of the blood vessel and is fundamental for endothelial cell survival and quiescence (23). Normal values of shear stress in medium-sized arteries range from 2.5 to 4.5 MegaPascal (MPa) (24).

In a Newtonian fluid, shear rate, shear stress and viscosity are linked due to subsequent the formula:

$$\eta =\frac{\tau }{\gamma }$$

Where η is the viscosity, τ the shear stress and γ the shear rate. When shear rate decreases viscosity increases and *vice-versa*. The presence of high shear rates (> 2,600 s^−1^ and even more >4,000 s^−1^) is typical of arterial stenosis and promotes endothelial damage, platelet adhesion and activation (25–28). When the shear rate is low, the flow profile flattens, and the various layers have similar velocities, then shear rate is extremely important for the risk of platelet activation and clotting factors because it is linked with shear stress and blood viscosity (25). Veins have low shear rates (<100 s^−1^) but also left atrium under pathological conditions such as atrial fibrillation (28, 29). In these conditions, there may cause an increased risk of stagnation of blood due to its high viscosity predisposing to activation of clotting factors and platelets (28, 29).

Wall shear stress (WSS) stimulates the endothelial cells to secrete substances that promote vasodilation and anticoagulation. Low values are associated with endothelial dysfunction (30), atherosclerosis (31), and the formation and rupture of aneurysms (32). However, extremely high values can be associated with damage to the intimal layer (33), rupture of atherosclerotic plaques (33), formation and extension of dissections (34, 35), and the formation and rupture of aneurysms (32, 36). The Wall Shear Stress Gradient (WSSG), measured in Pascals per millimeter (Pa/mm), represents the spatial variation of WSS along the vessel wall. In other words, it reflects how rapidly the shear stress exerted by blood flow changes across the vessel surface (37). Significant fluctuations in WSS can induce non-uniform mechanical stress on endothelial cells, contributing to pathological processes. Positive WSS gradients have been associated with localized endothelial damage, promoting inflammation, the progression of atherosclerotic lesions, and complications such as vascular dissection and aneurysm rupture (37).

## Methods

### MRA imaging protocol

Carotid artery imaging was conducted using a 1.5 Tesla scanner (GE Healthcare, Milwaukee, WI, USA) equipped with a head–neck 8-channel phased array coil. Prior to positioning the patient, a 20-gauge cannula was placed in an antecubital vein and connected to an electronic power injector (Spectris, Medrad Inc., Pittsburgh, PA). The subject was positioned supine on the scan table and then advanced head-first into the magnet bore. Following the acquisition of scout images, a test bolus was employed to measure the transit time from the arm vein to the carotid arteries. Subsequently, high spatial-resolution contrast-enhanced magnetic resonance angiography (CE-MRA) was performed in the coronal plane, utilizing a fast spoiled gradient recalled-echo (GRE) sequence. The sequence parameters were set as follows: TR/TE, 4.6–1 ms; flip angle, 20°; matrix, 352×256; slab thickness, 1.2 mm; field of view, 36 mm; and voxel size, 1 × 1.4 × 1.5 mm.

The CE-MRA study was acquired with bolus tracking following the administration of 0.1 mmol/kg bodyweight of the gadolinium-based contrast agent (Gadovist), with an injection rate of 2 mL/s, followed by a 20 mL saline flush. The pre-contrast data set was subtracted from the post-contrast data set to eliminate background signals. The subtracted data set was then utilized to generate maximum intensity projection (MIP) images of the entire carotid circulation.

### Extraction of MRA images and modeling

**Figure 2:**
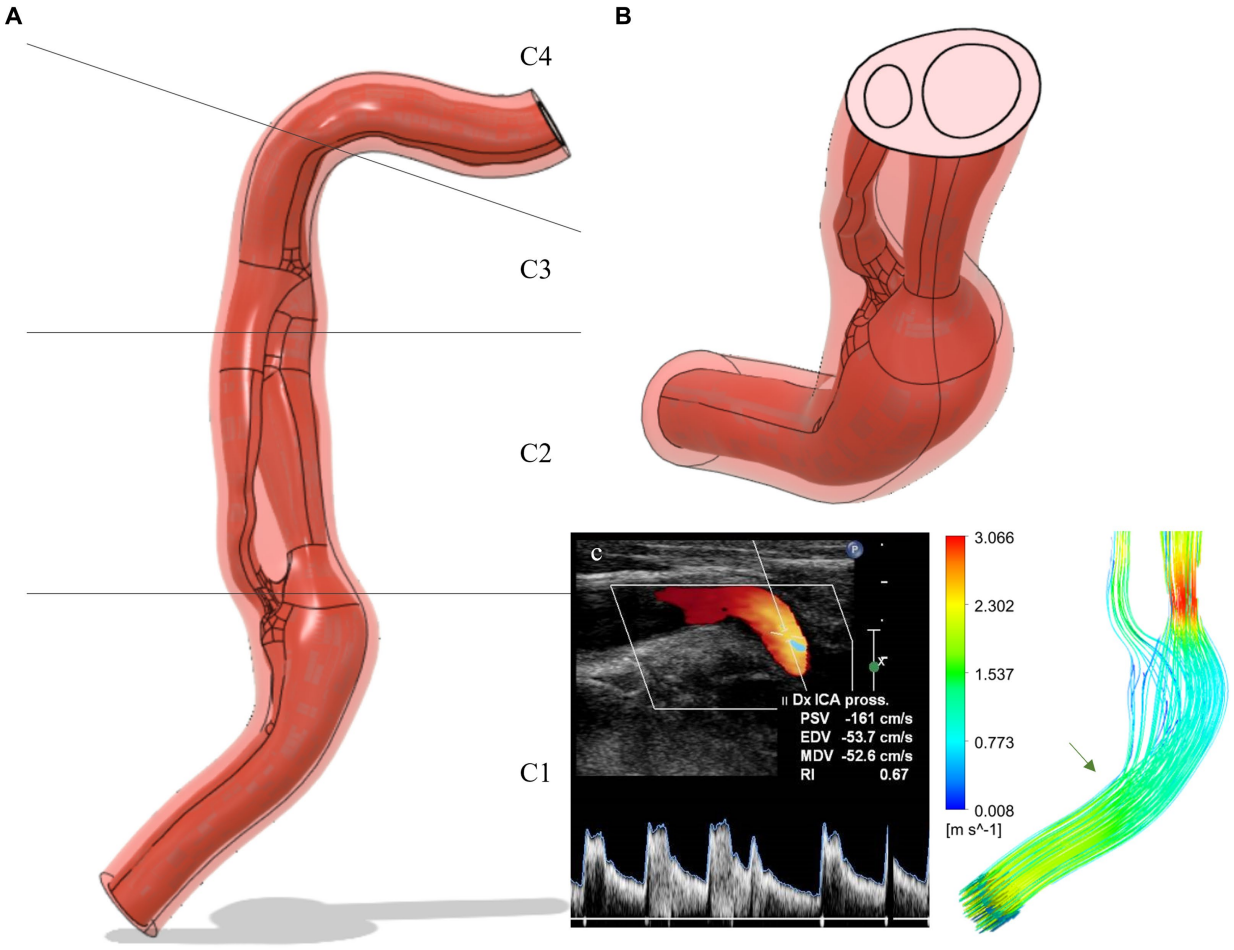
**(A)** Carotid dissection 3D-model extracted from MRI data (frontal view): flux in red and vessel wall in light red. The C1, C2, C3, C4 segments are shown in detail. **(B)** Cut-plane of the model: the double lumen separated by the intimal flap can be observed. **(C)** Simulation of flow velocity and comparison with Doppler data: the green arrow indicates the site of vessel kinking corresponding to the Doppler images. ICA, internal carotid artery; Dx, Right; Pross, proximal; PSV, peak systolic velocity; EDV, end-diastolic velocity; MDV, mean diastolic velocity; RI, resistivity index.

The GRE-MRA 3D sequences of the patients were imported into the open-source software 3D Slicer (v. 4.10, The Slicer Community) in DICOM format. The Level tracing function was employed to analyze the signal intensities of the contrast medium that flows in the blood vessels. This analysis revealed an intensity threshold within the range of 99–577. Subsequently, the scissor function was used to select only the right ICA from C1 segment to the C4 segment, eliminating other blood vessels. Smoothing was applied with a Kernel size of 2.00 mm. The model was then exported as an STL file and subsequently modified using CAD modeling software MeshMixer (v 3.5, Autocad) (38). After correcting the geometry, the file was imported into Rhinoceros 7.0 software, and based on the MRI information, the vessel wall was reconstructed through an offset operation. The ratio between wall thickness and vessel diameter was set to 1/8, in accordance with the value observed using Doppler on this patient. The models were then transformed into STEP files and imported into the multi-physics modeling software Ansys (v18.1 Ansys, Inc.) (16) (Figure 2).

### Biological tissues modeling

The properties of the vessel wall and fluid were assigned according to physiological characteristics. In our model, the wall was simplified and considered as an isotropic material linear elastic (16). To simulate incompressibility, a Poisson’s ratio of 0.49 was set. Two different Young’s moduli (1.5 MPa, 4 MPa) were assigned to the wall to simulate a normal or atherosclerotic internal carotid artery (39).

Human blood was simulated as a Newtonian fluid model with a density of 1.062 kg/m^3^ and a viscosity of 0.0037 Pa*s to replicate the properties of human blood at high shear rate values (16).

### Computational analysis

A computational approach of two-way fluid–structure interaction (FSI) was adopted, where information is exchanged on the interface surface between the fluid and the wall, transferring from the structure to the fluid and vice versa. Two domains were created: a structural domain for the vascular wall and a fluid domain for blood flow. For the structural part, a Transient Structural analysis was set up. The vessel was constrained at the ends without any imposed load, although the vascular wall is physiologically associated with intrinsic contractility. The fluid domain was modeled using the computational fluid dynamics software CFX (v18.1 Ansys, Inc.) (40).

The following boundary conditions were set: a pressure curve directly applied to the inlet, corresponding to the pressure variation between systole and diastole during the cardiac cycle. The applied pressure curve follows the data extracted from the invasive measurement of carotid arterial pressure (41). Three different pressure regimes were simulated: hypotension with a systole of 100 and diastole of 60, normotension with a systole of 120 and diastole of 80, and high pressure with a systole of 160 and diastole of 100. At the outlet, a static pressure was set to be 10 mmHg lower than the diastolic pressure of the corresponding regime, consistent with the pressure distribution analyzed in the intra- and extracranial cervical arteries (42). The two computation blocks were coupled using the System Coupling calculation code, which is also integrated into the Ansys Workbench calculation suite (40). The simulation completed more than six cardiac cycles, each lasting 0.86 s (approximately 70 bpm). After the results stabilized, it was deemed appropriate to utilize the third cycle, as the cyclic behavior of the curves was confirmed in subsequent cycles.

### Pipeline of the computational analysis

The results of the fluid dynamics study were analyzed in terms of shear strain rate, flow velocity, intraluminal pressure, wall shear stress (WSS), and wall shear stress gradient (WSSG). Shear strain rate, flow velocity, intraluminal pressure and WSS was obtained as putting them as output in Ansys.

WSSG (Pa/mm) was obtained analyzing the shear rate gradient at the vessel wall in Ansys, multiplied by viscosity. The structural analysis results were examined in terms of vascular wall deformation.

Specific points in the internal carotid geometry were referenced using radiological nomenclature, namely sections C1, C2, C3, C4: (1) The cervical segment runs from above the carotid bulb through the neck to the base of the skull; (2) the petrous segment runs from the base of the skull through the petrous bone; (3) the cavernous segment runs through the cavernous sinus (note the prominent bends); and (4) the supra-clinoid segment runs above the clinoid process through the dura into the subarachnoid space (43).

The model’s validation was conducted by analyzing flow velocities under normal blood pressure conditions and with a non-atherosclerotic vascular wall, comparing the results with actual patient data obtained from Doppler ultrasound. This condition was chosen because it closely matched the patient’s blood pressure data and the thickness of the intima-media layer, which serves as a marker of systemic atherosclerosis (44). Subsequently, the results were analyzed and compared under six different conditions: hypotension, normotension, and hypertension with a normal vascular elasticity regime, as well as hypotension, normotension, and hypertension with an atherosclerosis regime. The obtained results were subsequently used to analyze the risk of thrombosis, the risk of wall rupture or deformation, the risk of cerebral hypoperfusion, and the risk of dissection progression. The risk of thrombosis was assessed using shear strain rate values. Dissection progression risk was evaluated by analyzing WSS values in the terminal portion of the intimal flap, as several studies have demonstrated an association with intimal flap progression (34, 35). Deformation and rupture risk were analyzed using WSSG values. Furthermore, we assessed the movement of the intimal flap and the presence of pressure gradient between the true and false lumen.

Vessel and patient forecasted data, derived by computational analysis, were compared to dissection progression, artery wall dilatation and patient’s clinical and radiological data recorded at 6-month follow up visit.

### Statistical analysis

Quantitative variables were presented as means (and standard deviations), while categorical variables were expressed as numbers and percentile values. The analysis covered all explored conditions and specific vascular regions (see Table 1).

**Table 1:** Fluidodynamic and mechanical parameters.

Systole											Diastole								
	V_max_ (cm/s)	V_min_ (cm/s)	SR_max_ (s^−1^)	SR_min_ (s^−1^)	P_max_ (Pa)	P_min_ (Pa)	ΔP (Pa)	WSS_max_ (Pa)	WSS_min_ (Pa)	Deformation_max_ (mm)	V_max_ (cm/s)	V_min_ (cm/s)	SR_max_ (s^−1^)	SR_min_ (s^−1^)	P_max_ (Pa)	P_min_ (Pa)	WSS_max_ (Pa)	WSS_min_ (Pa)	ΔP (Pa)
Localization	C2 true lumen	C1 false lumen	C2 true lumen	C2 false lumen	C1 terminal part	C4 middle part	C2 between true and false lumen	C2 true lumen	C2 false lumen	C3 medial wall	C2 true lumen	C1 false lumen	C2 true lumen	C2 false lumen	C1 terminal part	C4 middle part	C2 terminal part of the intimal flap	C2 false lumen	C2 between true and false lumen
No aterosclerosis																			
Low pressure	314.2	0.1	1933.5	27.5	12957.3	8273.4	−4798.6	8.574	1.694	0.43	72.1	0	448.4	4.3	8273.4	8011	1.970	0.340	−262
Normal pressure	309	0.5	1914.2	33.6	15176.2	10467.9	−4695.9	8.477	1.790	0.53	101.7	0	632.5	9.1	10467.9	9710.5	2.789	0.495	−519.8
High pressure	409	0.7	2516.3	57.9	21123.7	13503.1	−8203.8	11.184	1.803	0.69	112.9	0.1	700.1	14.1	13503	12621	3.085	0.496	−8203.8
Aterosclerosis																			
Low pressure	313.1	0.5	1966.3	20.8	12925.2	5361	−4981.3	8.750	1.810	0.15	72.7	0	454.5	5.7	8330.2	8005.1	1.999	0.250	−271
Normal pressure	308.7	0.2	1964.7	31.5	15143.4	7363.8	−4702.4	8.841	1.856	0.20	102.7	0	642.3	9.7	10466.2	9696.4	2.827	0.536	−524.3
High pressure	422.4	0.1	2786.3	54.7	21130.8	7826.6	−8763.3	11.292	2.196	0.24	113.8	0	709.2	13.5	13501.7	12607.3	3.120	0.540	−640.6

V, velocity; SR, shear rate; P, pressure.

Furthermore, a detailed analysis of shear rate was performed to determine the percentage of time spent above or below predefined threshold values (>2,600 s^−1^ and < 10 s^−1^) during the cardiac cycle (see Table 2). Statistical analysis was carried out using the open-source software JASP (JASP Team, 2022, Version 0.16.3 for Windows) (45).

**Table 2:** Shear rate during cardiac cycle in the points of maximum and minimum shear rate.

	Terminal part of the false lumen					Initial part of the true lumen			
	SR_max_ (s^−1^)	SR_min_ (s^−1^)	Mean (s^−1^)	Time (s) < 10 s^−1^	Time (s) < 5 s^−1^	SR_max_ (s^−1^)	SR_min_ (s^−1^)	Mean (s^−1^)	Time (s) > 2,600 s^−1^
No aterosclerosis									
Low pressure	27.0	4.3	13.4 ± 7.2	0.33 (37.9%)*	0.05 (5.7%)	1933.5	360.8	1263.9 ± 560.0	0
Normal pressure	33.1	9.1	16.9 ± 7.8	0.03 (3.4%)*	0	1914.2	610.1	1253.6 ± 507.2	0
High pressure	58.2	14.1	31.9 ± 16.2	0	0	2516.3	657.3	1711.6 ± 714.3	0.07 (8.0%)*
Aterosclerosis									
Low pressure	23.0	5.7	12.1 ± 5.5	0.38 (43.6%)*	0	1966.3	355.2	1280.4 ± 575.4	0
Normal pressure	32.4	9.7	17.8 ± 8.4	0.03 (3.4%)*	0	1964.7	640.4	1355.2 ± 540.4	0
High pressure	51.8	13.5	27.8 ± 14.2	0	0	2786.3	660.2	1783.9 ± 753.6	0.13 (14.9%)*

* cardiac cycle time percentage. SR, shear rate.

## Results

### Model validation

The model validation was performed by comparing the flow velocities analyzed by Doppler with those evaluated in the simulation. The pressure regime of the simulation was normotensive (systolic 120/80 mmHg) as during the Doppler examination, the patient’s blood pressure was 126/85 mmHg. The elastic regime for the validation was a non-atherosclerotic wall since the patient exhibited an intima-media thickness (IMT) of 0.73 mm. This parameter serves as a marker of atherosclerosis and cardiovascular risk when the values exceed 0.9 mm (44). The point of velocity analysis was in the middle C1 segment of the right internal carotid artery. The velocities evaluated by Doppler were peak systolic velocity (PSV) = 161 cm/s and end diastolic velocity (EDV) = 53.7 cm/s and they resembled those of the simulation, in the same point, with the velocity during systole of 152.9 cm/s, while during diastole of 49.8 cm/s (Figure 2C).

### Flow velocity analysis

**Figure 3:**
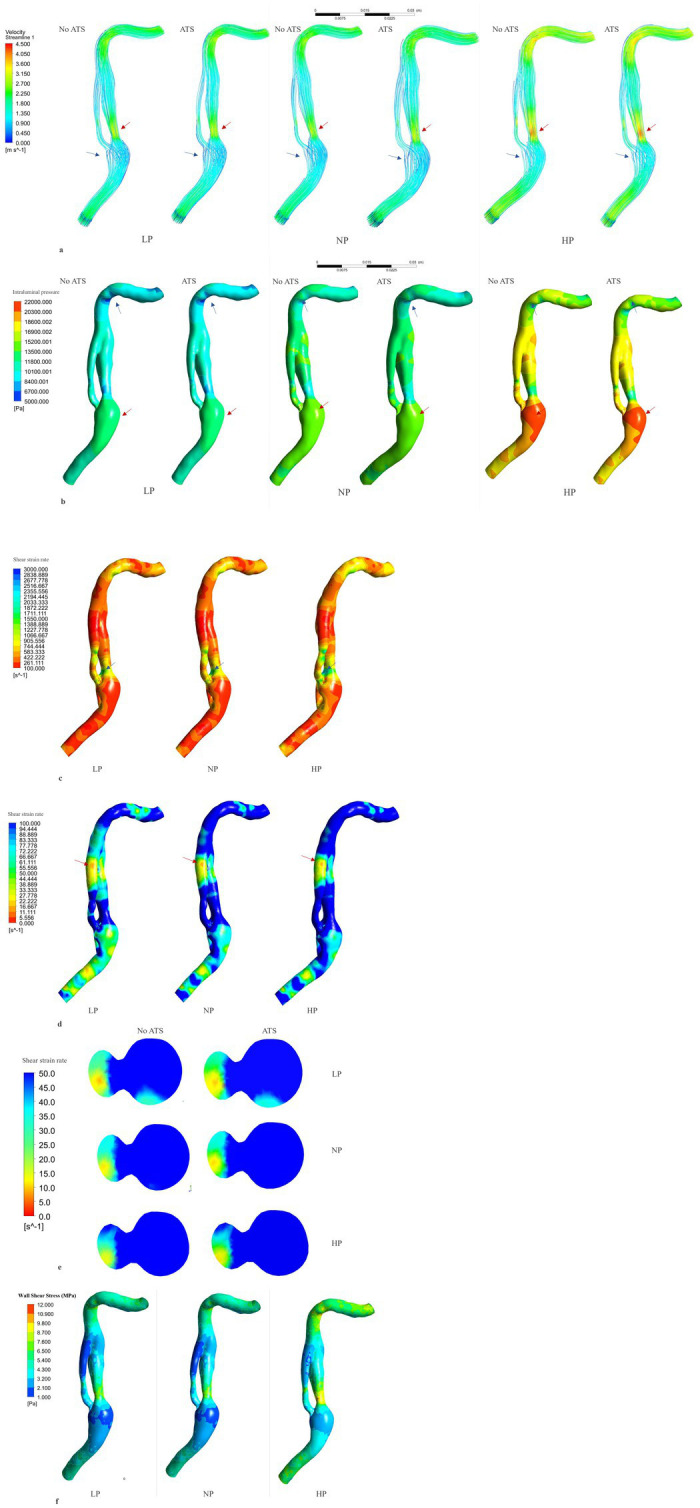
Flow velocity, intraluminal pressure, shear rate and shear stress analysis. Flow velocity analysis (systolic phase) **(A)**: the red arrows indicate the point of higher velocity, while the blue arrows indicate the point of lower velocity. The region of higher velocity is observed in the proximal segment of the dissection, specifically in the true lumen, where it can exceed 400 cm/s under hypertensive conditions. Conversely, the region of lower velocity is found near the intramural hematoma, where the fluid flow is relatively low. Intraluminal pressure analysis **(B)** (systolic phase): the red arrows indicate the point of maximum pressure, in the vessel bulge. The blue arrows indicate the point of minimum pressure, in the vessel kinking in C4. Shear rate > 100 s^−1^ analysis **(C)** (non-atherosclerotic conditions, systolic phase). The maximum shear rate is reached in the lateral face of the initial part of the true lumen (blue arrow) with peak values under hypertensive and atherosclerotic conditions (2786.3 s^−1^). Shear rate < 100 s^−1^analysis **(D)** (non-atherosclerotic conditions, diastolic phase). The minimum shear rate is reached in the distal part of the false lumen (red arrow) with lower values under hypotensive and non-atherosclerotic condition (4.3 s^−1^). Minimum shear rate, transversal section of the distal part of the dissection **(E)** (diastolic phase). Wall shear stress analysis **(F)** (non-atherosclerosis condition and systole phase). Highes values are reached in hypertension condition in the initial part of the true lumen. ATS, atherosclerosis; LP, low pressure; NP, normal pressure; HP, high pressure; No ATS, no atherosclerosis.

The Figure 3A and Table 1 present data on velocity in three pressure regimes and two elastic conditions of the vessel wall. The maximum velocity is reached at the proximal C2 segment, in the true lumen immediately after the beginning of the dissection (red arrow in the Figure 3), with maximum values in conditions of arterial hypertension and atherosclerosis (V_max systolic_ = 422.4 cm/s, V_max diastolic_ = 113.8 cm/s). Another point with high velocity is located in the C4 portion where the vessel kinks (Figures 3A–C). On the contrary, the minimum velocity is found in the proximal portion of the false lumen, after intramural hematoma at the distal C1 segments, with values close to zero in all analyzed conditions (blue arrow in the Figure 3A).

### Intraluminal pressure analysis

**Figure 4:**
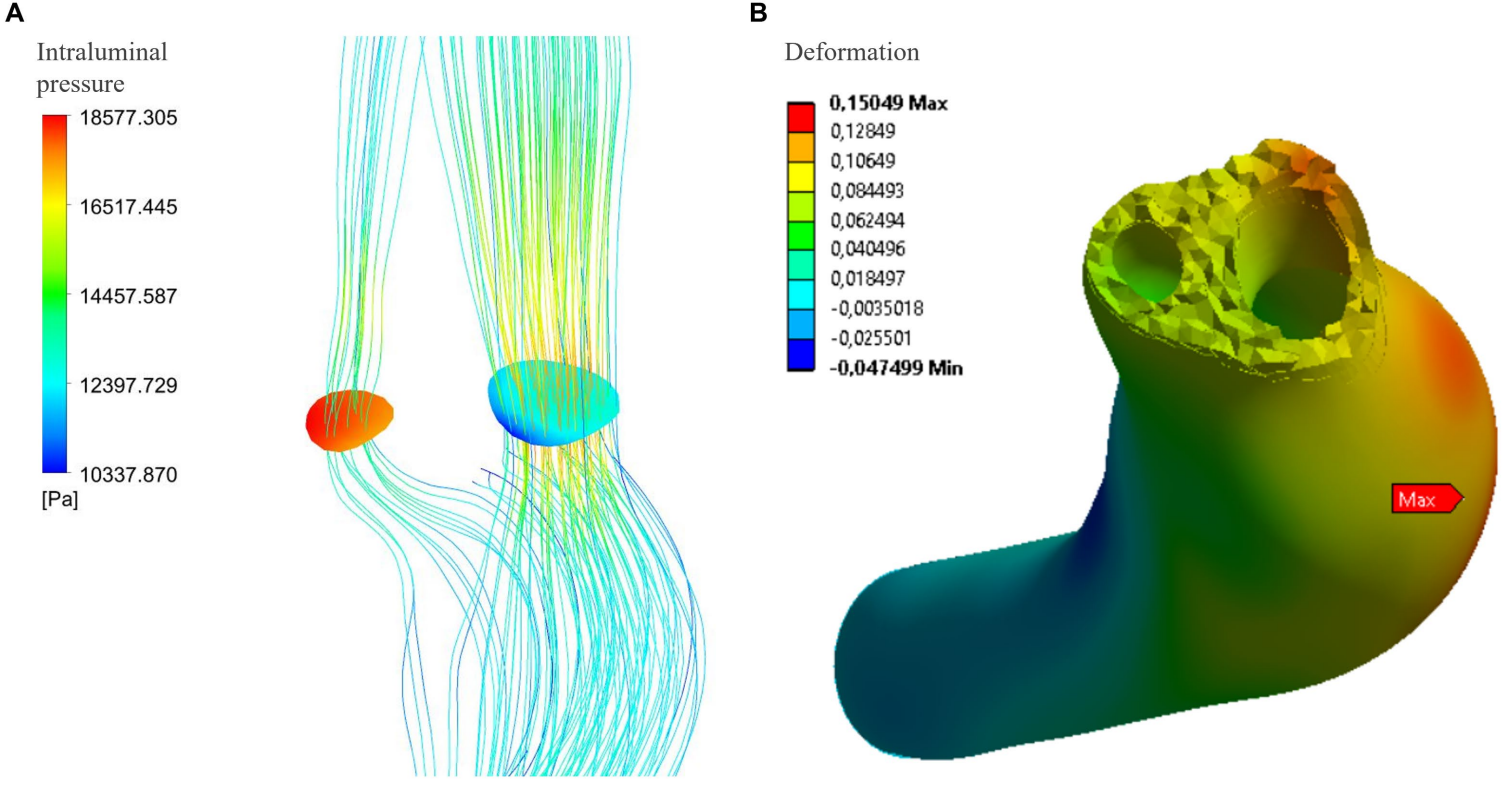
**(A)** Maximum delta pressure between true and false lumen (systolic phase): under conditions of atherosclerosis and arterial hypertension, the pressure difference reaches 8204.1 Pa. However, this pressure delta is not associated with any movements or deformations of the intimal flap in the structural analysis of the vessel wall at that specific location **(B)**.

The Figure 3B and Table 1 show the intraluminal pressure data in the different conditions analyzed. The maximum intraluminal pressure was reached before dissection, at the vessel bulge, medially to the intramural thrombus (Figure 3B red arrow). The maximum value was recorded during systole, under conditions of arterial hypertension and atherosclerosis (P_max_ = 21190.8 Pa). The elasticity of the vessel wall had a negligible effect on overall pressure values (Table 1). The minimum pressure was reached at the point of vessel bending in C4 during systole and under conditions of hypotension and atherosclerosis (*P*_min_ = 5,361 Pa). Another point with low pressure was located in the initial portion of the true lumen, also during systole and under conditions of hypotension and atherosclerosis (Figure 4). In this portion, there was a pressure delta between the true (at a lower pressure) and false lumen (at a higher pressure), which is maximum under conditions of arterial hypertension and atherosclerosis during systole (ΔP = −8204.1 Pa) (Figure 4A).

### Shear strain rate analysis

**Figure 5:**
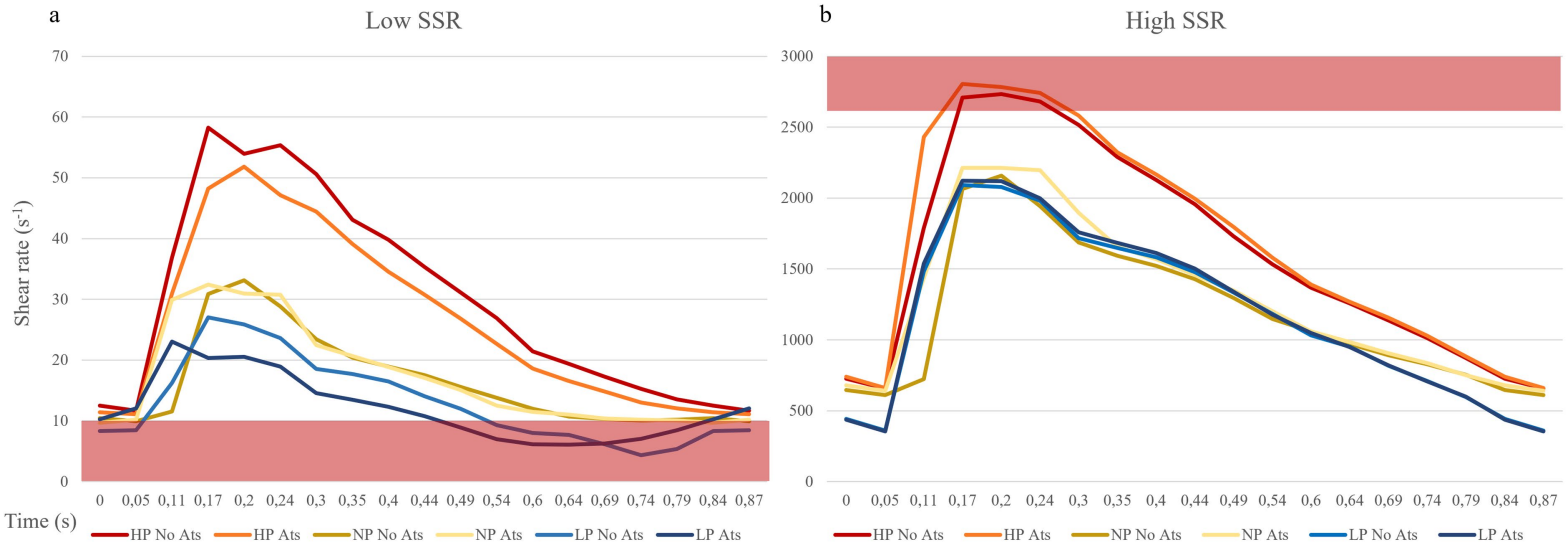
Shear rate curves during the cardiac cycle. **(A)** Point of lowest shear rate: the red bar indicates the shear rate values cut off for high thrombosis risk (10 s^−1^). **(B)** Point of highest shear rate: the red bar indicates the shear rate values cut off for mild thrombosis risk (2,600 s^−1^). ATS, atherosclerosis; LP, low pressure; NP, normal pressure; HP, high pressure.

The shear strain rate was maximum in the initial portion of the true lumen, at the point of maximum flow velocity (Figures 3C,D). The values depended strictly on arterial pressure, exceeding the value 2,600 s^−1^ under conditions of systole, arterial hypertension and atherosclerosis (SR_max_ = 2786.3 s^−1^). Stable values higher than 2,600 s^−1^ were found for 0.189 s (21.7% of the cardiac cycle) under this condition. Atherosclerosis determined slightly higher maximal values of maximal shear rate in all pressure regimes, leading to an increase of 4.1% in the hypertensive regime, 7.8% in the normotensive regime, and 1.1% in the hypotensive regime, with almost identical curves (Figure 5 and Table 1). The minimum shear strain rate was found at the end of the false lumen (Figures 3C,E), with stably <60 s^−1^ values in all conditions studied. Pressure was the major determinant of minimum shear strain rate values, leading to values <10 s^−1^ for about 0.360 s in the hypotensive regime (41.3% of the cardiac cycle), both under normal and atherosclerotic arterial wall conditions. In the normal pressure regime, values were = 10 s^−1^ for 0.23 s (26.4% of cardiac cycle) both under atherosclerotic and normoelastic conditions. Shear rate values <5 s^−1^ were reached in the hypotensive regime, both under atherosclerotic and normoelastic wall conditions for a few tenths of a second. Atherosclerosis determined an 8.3% reduction in average shear rate values in the hypotensive regime, an increase in average values of 4.1% under normotensive conditions, and a reduction of 12.8% under conditions of arterial hypertension (Figure 5 and Table 1).

### Analysis of the wall shear stress

**Figure 6:**
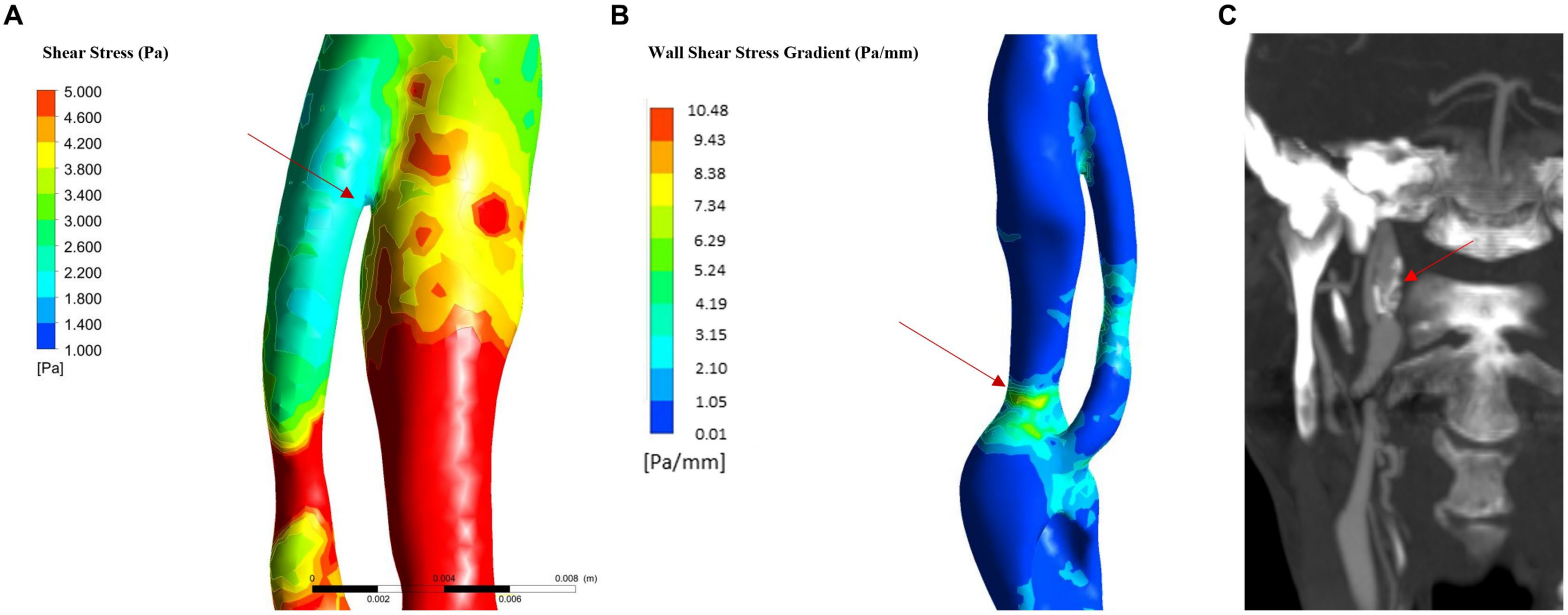
WSS in flap and WSSG analysis (in hypertension and atherosclerosis). **(A)** WSS in the last part of intimal flap: the analysis showed low WSS in the terminal part of the intimal flap. **(B)** WSSG: the maximum value is reached in the posterior part of the true lumen in the C2 proximal segment; **(C)** 6-month follow up CTA show carotid dilatation in C2 proximal segment as predicted by WSSG analysis.

The physiological shear stress in medium-sized arteries ranges from 2.5 to 4.5 Pa during systole (24). The maximal values of wall shear stress (WSS) were found at the initial segment of the true lumen, ranging from 1.9 to 3.1 Pa during diastole and from 8.5 to 11.3 Pa during systole (Table 1 and Figure 3F). Higher pressure conditions and the presence of atherosclerosis were associated with increased WSS values. The minimal values of WSS were observed at the terminal portion of the false lumen, ranging from 1.7 to 2.2 Pa during systole and from 0.3 to 0.5 Pa during diastole (Table 1 and Figure 3F). In order to assess the risk of aneurysm formation, the WSSG (WSS gradient) was calculated, which showed highest values in the initial part of the true lumen medial wall (max value in hypertension and atherosclerosis condition = 10.48 Pa/mm—Figure 6C). Moreover, we analyzed the WSS value in the terminal segment of the intimal flap since it is associated with risk of cranial extension of arterial dissection (34, 35). At this point, the values ranged from 0.4 to 0.5 Pa during diastole and from 1.7 to 2.2 Pa during systole in hypertension and atherosclerotic condition, that are comparable to physiological values (Figure 6A) (24).

### Analysis of wall deformation

The analysis of the wall deformation allows to evaluate the presence of the intimal flap movements predisposing to functional stenosis or occlusion of the true lumen. The analysis did not show significant intimal flap deformation or movement. The point that undergoes the greatest deformation (overall deformation in the three axes) is found at the medial wall of the vessel in segment C3 and is maximum under normal elastic and normotensive conditions (*d* = 0.53 mm). This deformation is caused by the systolic blood pressure exerting a force on the vessel so that it curves at that point. Directional deformation along the x axis (transverse deformation) showed the highest values in the postero-medial wall of terminal segments C1 and C2.

## Discussion

We developed a numerical model using FSI analysis of neuroradiological data from a patient with symptomatic carotid dissection. The aim was to enhance our understanding of the factors influencing the onset of neurological symptoms and to forecast the clinical and radiological progression of the dissection.

### Understanding stroke pathophysiology

Our investigation began with the observation that our patient consistently exhibited symptoms during episodes of elevated blood pressure. These ischemic symptoms may be secondary to temporary hypoperfusion or by micro-embolic events from artery-to-artery embolization. This analysis allowed us to define that the ischaemic symptomatology seems to be related more to a hypoperfusion mechanism than a thrombotic mechanism.

Indeed, we deduced that this occurrence might be attributed to the movement of the intima layer, resulting in the occlusion of the true lumen and causing temporary hypoperfusion in the right hemisphere. Although our simulated model indicated the absence of observable flap movements, an examination of intramural pressure unveiled a significant pressure disparity between the true and false lumen in the initial phase of the dissection. In this segment of the vessel, there is a narrowing occurring specifically in the true lumen, leading to an acceleration of flow and a decrease in intramural pressure in line with the Venturi effect (46) (decrease in the intraluminal pressure whit an increase flow velocity). The pressure difference increases with higher blood pressures and the presence of atherosclerotic plaques, as both are associated with an increase in velocity in the stenotic tract (Figure 3A). This mechanism could have mechanical implications in dissections characterized by an intimal flap, as the presence of different pressure values between the true and the false lumen could lead to a pressure gradient and movement of the flap itself (47). The reason why such movement does not occur in our model could be due to the larger thickness of the flap in that part of the dissection model than in the real artery, that lead to lower possibility of movement. Furthermore, to model the vascular wall, a cumulative Young’s modulus was used, which considers the wall as composed of all three vascular layers. The flap consists only of the vessel’s intima, and therefore, it is associated with a lower elasticity modulus than that simulated. On the other hand, the flap can be linked to thrombotic and calcification phenomena that, *in vivo*, may lead to increased wall stiffness.

The analysis of the shear strain rate allowed to estimate the thrombotic risk of the dissection and define the pathogenetic mechanism underlying thrombus formation. When shear rate values reach 2,600 s^−1^, endothelial damage may occur and they may also trigger the exposure of von Willebrand factor (vWF) filaments (48, 49), and the rupture of atherosclerotic plaque with consequent distal embolism (33). The risk of platelet activation and adhesion, is even higher for a shear rate above 4,000 s^−1^ due to the maximal unfolding of the vWF (28, 29). In addition, not only the value of the shear rate but also the duration of platelet exposure to a high shear rate or a sudden increase in the shear rate are important determinants (50). In the simulated model, we found highest shear rate values in the initial part of the true lumen only in condition of hypertension and severe atherosclerosis for <10% of cardiac cycle (Figure 5 and Table 2).

Shear rate values <100 s^−1^ are commonly observed in venous flow regimes, while at values below 50 s^−1^, the viscosity of plasma increases, leading to blood stasis (22, 26, 51). In such regimes, the risk of thrombosis increases leading to activation of coagulation factors, erythrocytes aggregation, and formation of rouleaux (52) and platelet aggregation, which occurs through the activation of coagulation factors, fibrinogen, glycoprotein IIb/IIIa, and glycoprotein IV (28, 29). Even if determining a precise lower cut-off limit for thrombotic risk is complex (22, 28), it is worth noting that sustained shear rates below 10 s^−1^ can significantly increase the risk of thrombosis (16) as they are commonly observed in conditions such as venous insufficiency and atrial fibrillation (16). In our model, the point of lowest shear rate was found at the level of the terminal part of the false lumen with values below 60 s^−1^ in all pressure regimes and elastic conditions studied. Values lower than critical value of 10 s^−1^ are observed in the hypo- and normotensive regimes. It is important to note that, only under hypotensive conditions, shear rate values consistently remained below the critical threshold (for 40% of the cardiac cycle), leading to a significant increase in thrombotic risk.

Our simulation shows that the most important risk factor for thrombosis is arterial pressure, as it influences blood flow velocity and thus shear rate values. Atherosclerosis may cause small variations in shear rate values, with slight increase in high shear rate conditions and decrease in low shear rate conditions. Simulation analysis showed that the greatest risk of thrombosis in this patient was associated with extremely low shear rates regimes rather than extremely high shear rates then thrombosis and it may occur in the terminal part of the false lumen due to blood stasis and activation of clotting factors. Furthermore, the risk of embolism and ischemic stroke appears to be plausible only under prolonged periods of hypotensive pressure conditions so not correlated with the clinical history of the patient. Other ischemic causes, such as the presence of a carotid warning syndrome in the context of small vessel disease (SVD), could potentially explain the patient’s symptoms. However, it is difficult to rule out these causes entirely, although they seem unlikely due to the size of the lesion (>1.5 cm^2^) and the low Fazekas score, which makes the presence of significant SVD less probable.

### Prediction of dissection evolution

Numerical simulation allowed us to analyze of the potential evolution of the dissection in terms of risk of thrombosis, extent of dissection, risk of vessel occlusion and risk of wall deformation.

The simulation results were compared with the patient’s clinical and imaging data at the 6-month follow-up. Firstly, it was necessary to find the most appropriate blood pressure and elastic regimen that matched with the patient’s clinical characteristic. At discharge, the patient was instructed to keep a blood pressure diary showing average blood pressure values of about 130/90 mmHg. To determine the most appropriate elastic regimen, intima-media thickness was measured by ultrasound, yielding values of 0.72 mm, which is below the cut-off for atherosclerosis. The clinical data were therefore compared with the normotensive and normo-elastic regimes. The presence of well-controlled blood pressure values reduced the risk of movements of the intimal flap. Furthermore, the presence of wall calcifications in Figure 1E has led to an increase in the elasticity modulus and a reduction in the likelihood of movement. The predicted risk of thrombosis is extremely low with this system, as the shear rate remains mostly <2,600 s^−1^ and > 10 s^−1^ throughout the cardiac cycle. The patient’s clinical and radiological data at 6 months confirmed the predictions of the simulation and showed no new ischaemic events. However, it is important to consider the effect of antiplatelet therapy, which was initiated at discharge and helped to reduce the risk of thrombosis.

Shear stress analysis revealed a low risk of dissection extension. Several simulation studies of dissections have shown that high presence of elevated wall shear stress at the terminal part of the intimal flap are associated with an increased risk of dissection extension (34, 35, 53). Our analysis revealed WSS values within the normal range near the distal part of the intimal flap under all explored conditions then it was associated with a low risk of dissection extension. This finding is consistent with the results of the 6-month follow-up imaging, which showed stability of the dissection without any extension.

In addition, we evaluated the risk of pseudoaneurysm formation through the analysis of the WSS, that appears to be main parameter that correlate with vessel dilatation, and rupture (32, 36). Some studies showed that both very low and high wall shear stress (WSS) can be associated with the formation and/or rupture of aneurysms (32). WSSG showed higher diagnostic accuracy than the shear stress in formation and growth of aneurysmal dilatation of the vessel wall (36, 54). The WSSG represents the spatial gradient of the shear stress along the vessel wall and it increases in condition of flow acceleration as in the arterial stenosis (55). The analysis of WSSG showed predominantly high positive values at the level of the distal portion of the true lumen in all analyzed conditions. Zimny et al. paper show how a WSSG > − 0.32 Pa/mm is associated with an increased risk of aneurysm formation (AUC: 0.654) (36), so it is possible to infer the presence of a non-negligible risk of pseudoaneurysm development initial part of the true lumen in the medio-posterior wall of the segment C2 (Figure 6B). This result predicts the dilatation of the vessel wall and was consistent with the CTA findings at follow-up showing an aneurysm in this segment (Figure 6C).

The results of this study demonstrate that blood pressure is a critically important clinical factor in the management of carotid dissections. On one hand, it is essential to lower blood pressure to prevent the extension of the dissection, reduce the risk of vessel deformation, and avoid paradoxical occlusion of the true lumen. On the other hand, excessively low blood pressure can lead to cerebral hypoperfusion, increase blood stasis, and elevate the risk of thrombosis.

## Limitations

We acknowledge several limitations of our study. Our data derived by a single case observation and, because patient showed peculiar features, thus results should be replicated in a wider population. Moreover, the model incorporates several simplifications and limitations that may be misleading by real-world scenarios. Notably, the model simplifies blood vessels as isotropic, and linear elastic whereas in reality, they are anisotropic, non-linear, and incompressible materials (56, 57). Additionally, the model assumes a homogeneous vessel wall with a single material with a single Young’s modulus, whereas the actual blood vessel wall consists of three distinct layers (adventitia, media, and intima) with varying elastic properties. The chosen Young’s modulus in our model represents a composite value that accounts for the elastic contribution of all three layers omitting variations in their mechanical behavior (58). Such contractility could lead to variations in velocity, shear stress, and shear rate compared to real-world conditions. However, model validation revealed velocity values consistent with Doppler measurements, suggesting good approximation and accuracy in shear stress and shear rate values. Another limitation arises from the assumption that blood behaves as a Newtonian fluid, while human blood is a complex suspension of deformable and aggregable cellular elements in plasma, rendering it non-Newtonian in nature (59). Consequently, blood does not have linear behavior, as it registers an increase in viscosity values at low shear rates (SSR) (29, 60). Thus, regions with low shear rates in the model might exhibit lower viscosity than simulated.

## Conclusion

In conclusion, computational analysis may provide valuable insights into the pathophysiology of stroke in the case of a patient with ICA dissection. Moreover, simulation data demonstrated to resemble consistently with clinical and radiological 6-month outcomes. Future research should focus on the simulation of different types of dissections allowing us to analyze the effect of vascular geometry on the rheological characteristics of blood in different conditions.

## Data Availability

The datasets presented in this article are not readily available because of ethical and privacy restrictions. Requests to access the datasets should be directed to the corresponding author.
